# Influence of Novel Norovirus GII.4 Variants on Gastroenteritis Outbreak Dynamics in Alberta and the Northern Territories, Canada between 2000 and 2008

**DOI:** 10.1371/journal.pone.0011599

**Published:** 2010-07-16

**Authors:** Xiaoli L. Pang, Jutta K. Preiksaitis, Sallene Wong, Vincent Li, Bonita E. Lee

**Affiliations:** 1 Provincial Laboratory for Public Health (ProvLab), Edmonton, Alberta, Canada; 2 Department of Laboratory Medicine and Pathology, University of Alberta, Edmonton, Alberta, Canada; 3 Department of Pediatrics, University of Alberta, Edmonton, Alberta, Canada; 4 Department of Medicine, University of Alberta, Edmonton, Alberta, Canada; Washington University, United States of America

## Abstract

**Background:**

Norovirus GII.4 is the predominant genotype circulating worldwide over the last decade causing 80% of all norovirus outbreaks with new GII.4 variants reported in parallel with periodic epidemic waves of norovirus outbreaks. The circulating new GII.4 variants and the epidemiology of norovirus outbreaks in Alberta, Canada have not been described. Our hypothesis is that the periodic epidemic norovirus outbreak activity in Alberta was driven by new GII.4 variants evolving by genetic drift.

**Methodology/Principal Findings:**

The Alberta Provincial Public Health Laboratory performed norovirus testing using RT-PCR for suspected norovirus outbreaks in the province and the northern Territories between 2000 and 2008. At least one norovirus strain from 707 out of 1,057 (66.9%) confirmed norovirus outbreaks were successfully sequenced. Phylogenetic analysis was performed using BioNumerics and 617 (91.1%) outbreaks were characterized as caused by GII.4 with 598 assigned as novel variants including: GII.4-1996, GII.4-2002, GII.4-2004, GII.4-2006a, GII.4-2006b, GII.4-2008a and GII.4-2008b. Defining July to June of the following year as the yearly observation period, there was clear biannual pattern of low and high outbreak activity in Alberta. Within this biannual pattern, high outbreak activity followed the emergence of novel GII.4 variants. The two variants that emerged in 2006 had wider geographic distribution and resulted in higher outbreak activity compared to other variants. The outbreak settings were analyzed. Community-based group residence was the most common for both GII.4 variants and non-GII.4 variants. GII.4 variants were more commonly associated with outbreaks in acute care hospitals while outbreaks associated non-GII.4 variants were more commonly seen in school and community social events settings (p<0.01).

**Conclusions/Significance:**

The emergence of new norovirus GII.4 variants resulted in an increased norovirus outbreak activity in the following season in a unique biannual pattern in Alberta over an eight year period. The association between antigenic drift of GII.4 strains and epidemic norovirus outbreak activity could be due to changes in host immunity, viral receptor binding efficiency or virulence factors in the new variants. Early detection of novel GII.4 variants provides vital information that could be used to forecast the norovirus outbreak burden, enhance public health preparedness and allocate appropriate resources for outbreak management.

## Introduction

Norovirus (NoV) is recognized as the most common cause of gastroenteritis outbreaks worldwide. NoV outbreaks occur frequently in nursing homes, health care institutions, cruise ships and have also been reported in schools and prisons [1]–[3], causing considerable morbidity and mortality in these settings [4]. Noroviruses are extremely contagious with the minimal infectious dose as low as 10 viral particles [5] while the amount of virus shed by infected individuals is high (over 10^8^ RNA copies per gram of stool) [6]–[8]. Transmissibility is enhanced by the stability of NoV in the environment and its resistance to disinfection [9]. Norovirus can be transmitted by contaminated food and water, person to person spread, and exposure to aerosols from vomitus [10]–[12]. Norovirus outbreak is therefore a challenge to manage and control and has considerable health and economic impact.

Norovirus is a genetically diverse group of virus belonging to the family *Caliciviridae*. There are currently five genogroups (GI–GV). GIII has only been found in cattle [13], [14] and GV only in mice [15], [16]. GI (8 genotypes) and GII (17 genotypes) contain most of the strains infecting humans [16]. The NoV GII.4 genotype has been the predominant genotype circulating in the US, Europe and Oceania over the past decade and has caused up to 80% of all NoV outbreaks, particularly those occurring in healthcare settings [17]–[20]. Recent studies have reported that GII.4 capsid sequences have evolved over the last 20 years and in parallel, NoV outbreaks of epidemic proportion have been observed [21]. The pandemic spread of new GII.4 variants was first recognized in the mid-1990s [22]. In 1995–1996, strain US95/96 was responsible for about 55% of NoV outbreaks in the US and 85% in the Netherlands [23]. Between 2000 and 2004, US95/96 was replaced by two new GII.4 variants, the Farmington Hill strain [3], which was associated with 80% of the US NoV outbreaks [1]. During the same period in Europe, the new GII.4 variant caused outbreaks during the winter, spring and summer [24]–[26]. In 2004, the Hunter GII.4 variant was detected in Australia, Europe, and Asia [26]–[28]. This strain was then replaced by two co-circulating GII.4 variants in the US, Europe, and Asia in early 2006 [4]. Between 2007 and 2008, two new GII.4 variants emerged and were reported by the food-borne viruses in Europe (FBVE) Network (Joukje J., personal communication). All NoV GII.4 variants are genetically linked and have been classified internationally as novel GII.4 variants 1996, 2002, 2004, 2006a, 2006b and 2008 respectively.

The circulation of NoV GII.4 variants in Alberta and northern Territories in Canada and the epidemiology of gastroenteritis outbreaks has not been described. We examined nine years of gastrointestinal outbreak data (2000–2008) and studied the emergence and circulation of new GII.4 variants and their association with NoV gastroenteritis outbreak activity.

## Methods

### Ethics Statement

Health ethics was not requested as this study was part of routine laboratory-based outbreak investigation and identifiable information of the outbreaks was not included in the analysis. Patient consents were not required as they were tested as per routine outbreak laboratory investigations and patient demographic information was not included in the analysis.

### Laboratory investigation of gastroenteritis outbreaks

All suspected gastrointestinal outbreaks in the province of Alberta, Canada are identified and investigated through the provincial public health system using standardized protocols and tracking systems established since 1999. Between March 2004 and March 2009, the province was divided into nine health regions, including: two metropolitan regions each with a population >1 million and seven non-metro regions with populations ranging from 82.8 K to 183.5 K. The Alberta Provincial Public Health Laboratory (ProvLab) performed laboratory investigations on stool samples from all gastroenteritis outbreaks in Central and Northern Alberta between 1999 and February 2002 and for the whole province from March of 2002 onward. ProvLab also provided laboratory investigation support for gastroenteritis outbreaks in the northern territories in Canada (the Northwest Territories, Yukon and Nunavut) during this period. The settings of the outbreaks were recorded in ProvLab database and could be classified as one of the followings: group residence, senior residence, long term care, hospital long term care, hospital acute care, daycare, school, community gathering, conference, food establishment, cruise/hotel, private households, community shelter/service, region-based community and others.

If NoV was the suspected etiological agent for a gastroenteritis outbreak, stool specimens were processed for both standard bacterial culture and nucleic acid detection of NoV using conventional reverse transcript PCR between 1999 and March 2004 [29] and a multiplex real time RT-PCR (Mrt RT-PCR) assay since April 2004 [30]. At least one NoV positive specimen from each outbreak occurring between July 2000 and June 2008 was selected for genotyping by DNA sequencing.

### RT-PCR assay and Sequencing for Norovirus

The Mrt RT-PCR assay used for NoV detection in the stool specimens from gastroenteritis outbreaks and the methods for nucleotide sequencing to characterize NoV genotype and variants were previously described by Pang et al [30], [31]. Briefly, total nucleic acid from 200 µl of 10% stool suspension was extracted and eluted into 100 µl using Magazorb™ RNA extraction kit (CORTEX Biochem, CA, USA) by automatic extraction or easyMAG (bioMérieux) according to the manufacturer's instructions. cDNA was synthesized using random hexamer and SuperScript™ II RNase H^-^Reverse Transcriptase kit (Invitrogen, CA, USA). Five micro liters of cDNA was used for detection of NoV and the remaining cDNA sample was stored at minus 20°C for later characterization of NoV. cDNA samples tested positive for NoV was used for amplification with primers from region E of the capsid gene for GII and from region D for GI. If amplification failed with the region E primers for GII, then amplification was performed using the primers from region D for GII. The amplicons (GII region E = 320 bp, GII region D = 253, and GI region D = 177 bp) were purified using Qiagen purification kit (Qiagen, Chatsworth, CA, USA). Sequencing was performed using the BigDye® Terminator v3.1 Cycle Sequencing Kit (Applied Biosystems) in the ABI PRISM® 3100-Avant Genetic Analyzer. Sequencing analysis utilized the Data Collection Software v2.0 (Applied Biosystems).

### Phylogenetic Analysis

Primary sequence data was assembled and the consensus sequence from each amplicon was generated using the sequence software BioNumerics 5.1. The sequence data was blasted against the database (GenBank, NCBI) to confirm the NoV assignment before performing the phylogenetic analysis. Pairwise alignments and multiple alignments of DNA sequences were performed with the default parameters. A minimum spanning tree was constructed based on 320 nucleotides in the E region of the capsid gene using the neighbor-joining algorithm with BioNumerics software (version 5.1; Applied Maths Sint-Martens-Latem, Belgium).

Norovirus genogroup, genotypes and the GII.4 variants were assigned using the reference GenBank access numbers and nomenclature described by Zheng et al in 2006 [16], the FBVE network and CaliciNet, US (Dr. M Koopmans and Dr. J Vijin, personal communication) as shown in Table 1.

**Table 1:** Norovirus GII reference genotypes used in this study.

*GII genotypes	Name of strain	Accession number in GeneBank
GII.1	Hawaii/71/US	U07611
GII.2	Ina/02/JP	AB195225
GII.2	Snowmount/76/US	U75682
GII.3	Oberhausen455/01/DE	AF539440
GII.3	Toronro24/91/CA	U02030
GII.4	Lordsdale/93/K	X86557
GII.4	Bristol virus/94/UK	X76716
GII.5	Hillingdon/94/U	AJ277607
GII.5	Hokkaido133/03/J	AB212306
GII.6	Seacroft/90/UK	AJ277620
GII.7	Leeds/90/UK	AJ277608
GII.8	Amsterdam/98/NL	AF195848
GII.9	Virginia207/97/US	AY038599
GII.10	Erfurt-546/00/DE	AF427118
GII.11	Sw918/97/JP	AB074893
GII.12	Wortley/90/UK	AJ277618
GII.13	Fayetteville/98/US	AY113106
GII.14	M7/99/US	AY130761
GII.15	J23/99/US	AY130762
GII.16	Tiffin/99/US	AY502010
GII.17	CS-E1/02/US	AY502009

†GII.4 Variants	Name of strain	Accession number in GeneBank	Causing epidemic NOG worldwide
1996	DenHaag015/00-NL	AB303923	Before 2001
1996	Grimsby	AJ004864	Before 2001
2002	Farmington Hills/02/US	AY502023	2002–2003
2002	Haarlem457/2003-NL	AB303933	2002–2003
2004	0317/04/NL	AY883096	2004–2005
2004	Hunter 284E/04O/AU	DQ078794	2004–2005
2006a	Terneuzen70/06-NL	EF126964	2006–2007
2006a	Yerseke38/06/NL	EF126963	2006–2007
2006b	Nijmegen115/06/NL	EF126966	2006–2007
2006b	Minerva/06/UA	EU078417	2006–2007
2008a	OC07138/Jap	AB434770	Ongoing surveillance
2008b	Apeldoorn317/07/NL	AB445395	Ongoing surveillance

*Zheng et al. Virology, 2006.

† Personal communication from the Food-borne Viruses in Europe (FBVE) and Centers for Disease Control and Prevention (CDC) in US Network.

### Definition of the observation period, setting and epidemic year for NoV outbreaks

The annual observation period for NoV gastroenteritis outbreaks was defined as the period beginning in July and ending in June of the following year to improve the capture of the winter seasonality of NoV outbreaks. Norovirus outbreaks always peaked between the months of November to March. This seasonal assignment allowed better analysis of the annual fluctuations of NoV outbreak burden and its relationship to strain variation and evolution. For this study, an epidemic year was defined as when the number of NoV outbreaks during the 12-month period was ≥2 times higher than the numbers of outbreaks in the period before and after the observation period. The observation periods with a relatively low number of NoV outbreaks were designated as quiescent years.

For the analysis of the outbreak settings, outbreaks in group residence, senior residence and long term care were grouped together as community-based group residence. Outbreaks in community gatherings, conference and food establishments were considered as similar setting with sharing of food and beverages and were classified as community social events.

### Data Analysis

The difference in the number of NoV outbreaks by observation periods and the association of new variant strains and epidemic year was analyzed using binary logistic regression. Chi Square Test or Fisher Exact test as appropriate with Bonferroni correction was used to analyze the difference in NoV outbreaks for GII.4 variants and non-GII.4 variants in the following outbreak settings: community-based group residence, hospital long-term care, hospital acute care, community social events and school.

## Results

### Norovirus outbreaks and distribution pattern

**Figure 1:**
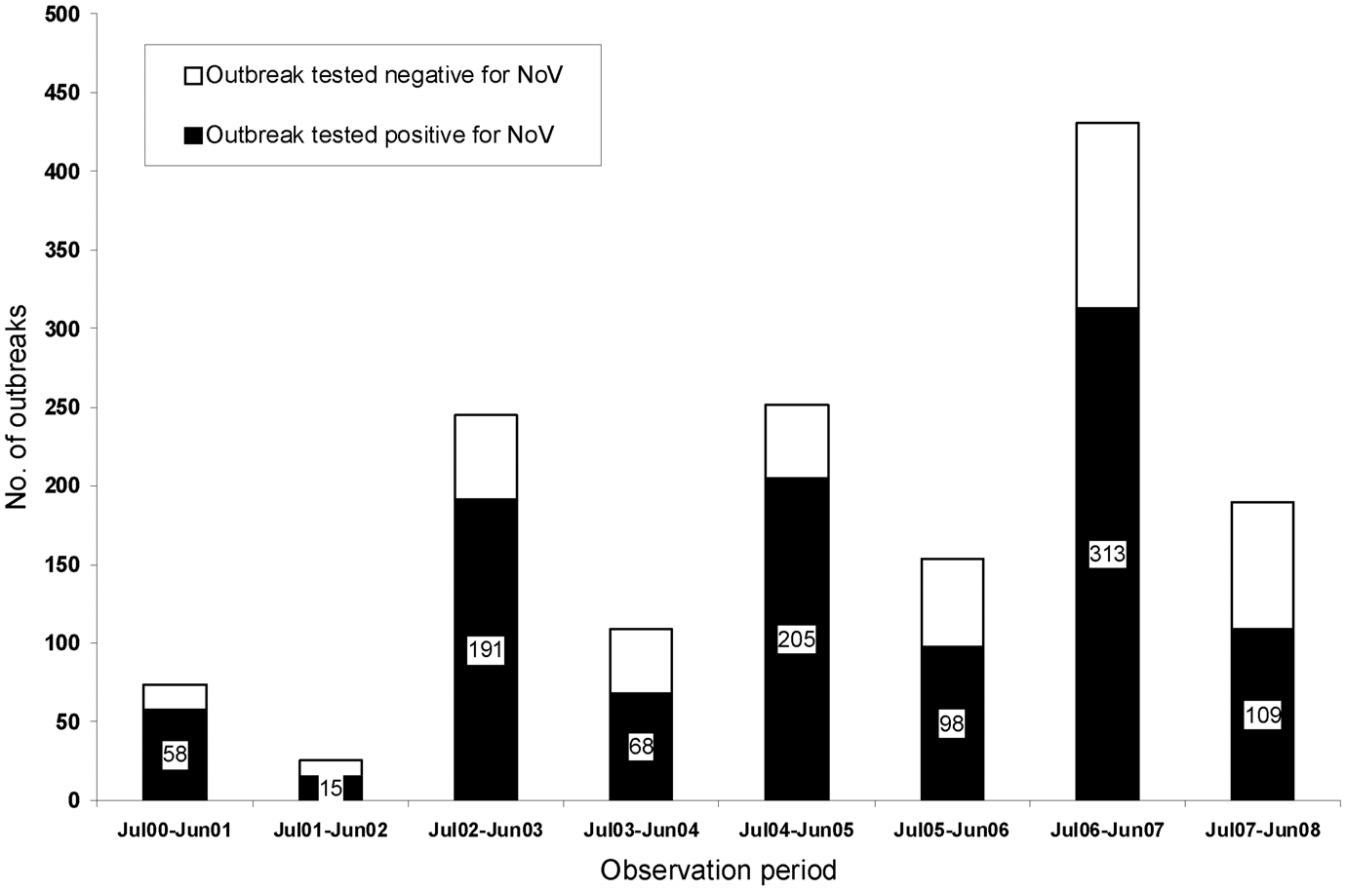
Number of norovirus outbreaks by observation periods from July 2000 to June 2008.

During the 8 year study period (July 1, 2000–June 30, 2008), stool samples from 1,480 gastroenteritis outbreaks were tested for NoV. Norovirus was identified as the causative agent in 1,057 outbreaks (71.4%). Confirmed NoV outbreaks demonstrated a clear alternating pattern of epidemic and quiescent years. Observed NoV outbreak activity per year (n = number of NoV outbreaks, % outbreaks NoV positive) was as follows during quiescent years: 2001–02 (n = 15, 57.7%), 2003–04 (n = 68, 64.2%), 2005–06 (n = 98, 63.6%) and 2007–08 (n = 109, 57.4%), and epidemic years 2000–01 (n = 58, 78.4%), 2002–03 (n = 191, 78.0%), 2004–05 (n = 205, 81.7%) and 2006–07 (n = 313, 72.6%), respectively (Table 2 and Figure 1). The proportion and number of gastroenteritis outbreaks caused by NoV was significantly higher during epidemic as compared to quiescent years (p<0.0001, binary logistic regression). This observation is independent of the variation in the number of NoV outbreaks in all eight observation periods. The number of NoV outbreaks observed increased over time in both epidemic as well as quiescent years during the study period.

**Table 2:** Norovirus genogroup and genotypes in outbreaks during July 2000 to June 2008.

Observation Period	Jul00 to Jun01	Jul01 to Jun02	Jul02 to Jun03	Jul03 to Jun04	Jul04 to Jun05	Jul05 to Jun06	Jul06 to Jun07	Jul07 to Jun08	Total
Number of norovirus outbreaks	58	15	191	68	205	98	313	109	1,057
Sequence (%)	21 (36.2)	3 (20.0)	77 (40.3)	29 (42.6)	158 (77.1)	56 (57.1)	268 (85.6)	95 (87.2)	707 (66.9)
G I total	0	0	2	16	0	1	9	2	30
G II total	21	3	75	13	158	55	259	93	677
GII.1	1				1				2
GII.2						10	1		11
GII.3	2	1	4	1	7	3	2	9	29
GII.4	18	2	71	10	148	41	252	75	617
GII.5					1			6	7
GII.6				2	1	1		3	7
GII.9							3		3
GII.13							1		1

### Norovirus genotypes

At least one NoV was successfully sequenced from 707 of the 1,057 confirmed NoV positive outbreaks (66.9%). The median sequencing rate was 57.1% (range 20.0–87.2%) for all observation periods. Analysis of the outbreaks with sequence data revealed 30 NoV genogroup (GI) outbreaks that occurred primarily in the 03–04 quiescent year (16/30, 53%) and the 06–07 epidemic year (9/30, 30%). Most of the NoV outbreaks were caused by NoV genogroup (GII) strains (n = 677). Genogroup II predominated in each observation period except for the period July 2003 to June2004. Among 677 NoV GII outbreaks, 617 strains were classified as GII.4 (91.1%), 29 as GII.3 (4.3%), 11 as GII.2 (1.6%), 7 each as GII.5 and GII.6 (1.0%). Norovirus GII.1, 9 and 13 were detected in less than 1% of outbreaks and no GII.7, 8 and 10 to 15 was found. Norovirus genotypes/strains and associated gastroenteritis outbreaks are summarized in Table 2.

### Temporal emergence of GII.4 variants and the epidemic years

**Figure 2:**
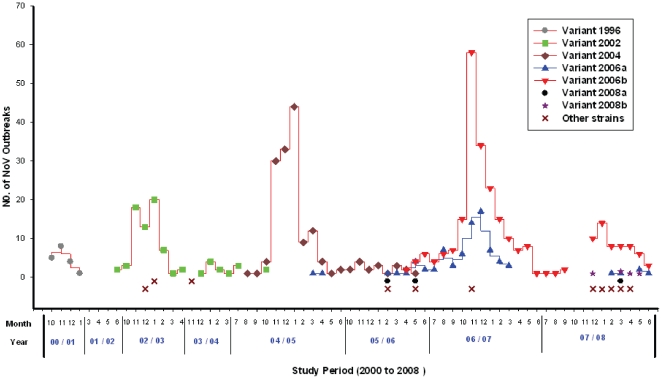
Emergence of new norovirus GII.4 variants from July 2000 to June 2008.

Between July 2000 and June 2008, six new GII.4 variants emerged in Alberta (Table 3). The variant 1996 was identified in the epidemic year 2000–01 and was the only NoV GII.4 variant causing outbreaks during this period. After a quiescent period for NoV outbreaks, a new GII.4 variant 2002 emerged in June of 2002 and caused a large number of NoV outbreaks in the epidemic year 2002–03. In the following quiescent period 2003–04, the variant 2002 continues to circulate but outbreak activity associated with this variant remained low (only 10 outbreaks). In the epidemic periods 2004–05 and 2006–07, the emergence of new variants 2004 (August 2004), 2006a (March 2006) and 2006b (April 2006) resulted in a pattern similar to that observed with 2002 variant. These new variants caused an even larger number of NoV outbreaks, (n = 141 in 2004–05 and n = 251 in 2006–07) respectively. Interestingly, while the 2006b and 2006a variants arose concurrently in the spring, the 2006b variant caused three times as many outbreaks as the 2006a variant: 189 and 62 outbreaks respectively. In the following quiescent period 2007–08, variant 2006b remained dominant even though both variants demonstrated decreased activity levels (Figure 2). The emergence of new variants was significantly associated with epidemic years (p<0.0001, binary logistic regression). The number of NoV outbreaks associated with the newly emerged GII.4 variants increased steadily in the four epidemic years between 2000 and 2007.

**Table 3:** Norovirus GII.4 variants in outbreaks during July 2000 to June 2008.

Observation Period	Jul00 to Jun01	Jul01 to Jun02	Jul02 to Jun03	Jul03 to Jun04	Jun04 to Jul05	Jul05 to Jun06	Jul06 to Jun07	Jul07 to Jun08
Number of norovirus GII outbreaks by sequence	21	3	75	13	158	55	259	93
Number of GII.4 variants outbreaks (%)	18 (85.7)	2 (66.7)	71 (94.7)	10 (76.9)	148 (93.7)	41 (74.5)	252 (97.3)	75 (80.6)
GII.4-1996	18			2				
GII.4-2002		2	61	8	5			
GII.4-2004					141	18		
GII.4-2006a					2	9	62	5
GII.4-2006b						12	189	61
GII.4-2008a								1
GII.4-2008b								4
GII.4-other			10			2	1	4

The 1996 variants were found in 4 of the 9 health regions in Alberta, the 2002 variant in 7 regions, the 2004 variant in 8 regions and an outbreak in Nunavut, the 2006a variant in 6 regions as well as a provincial outbreak and an outbreak in Yukon, and the 2006b variant in all 9 health regions in Alberta and an outbreak in the Northwest Territory. Both the 2002 and 2006a variant spanned four observation periods, with circulation observed between June 2002 and October 2004 and March 2006 to June 2008 respectively. The 1996 variant circulated from October 2000 to January 2001 and reappeared in November of 2003.

**Figure 3:**
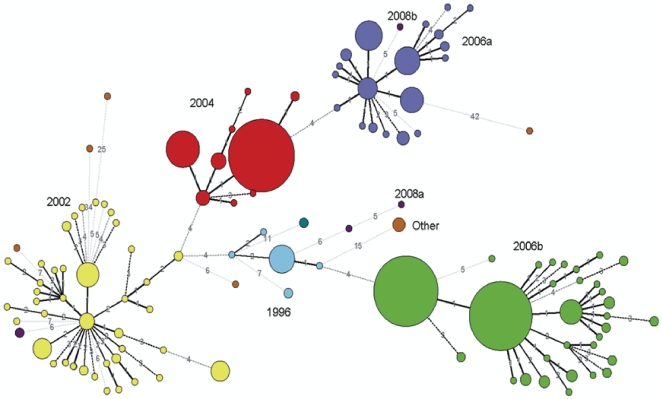
Phylogenetic tree of region E of the norovirus capsid. This figure is generated by using the neighbor-joining algorithm with BioNumerics software (version 5.1; Applied Maths Sint-Martens-Latem, Belgium).

Alignment analysis on 320 nucleotides in the E region of the capsid gene demonstrated the genetic distances between newly emerging variants and the previous ones. Most of the variants seemed to have evolved from their antecedent temporal variants with a varying number of nucleotide changes, as illustrated by comparing variant 2004 to variant 2002. However, genetic lineages of the variant 2006b and 2006a were completely different, with the 2006b variant linked closely to the1996 variant and the 2006a variant to the 2004 variant. Interestingly, both strains were associated with NoV outbreaks in the same epidemic year (Figure 3).

### Clinical settings of GII.4 variants and non-GII.4 variants outbreaks

The three most common settings for outbreaks caused by GII.4 variants were community-based group residence (n = 427, 69.2%) followed by hospital long-term-care (n = 92, 14.9%), hospital acute care (n = 44, 7.1%). The three most common settings for non-GII.4 variant outbreaks were community-based group residence (n = 56, 62.2%), followed by schools (n = 8, 8.9%) then community social events (n = 7, 7.8%) (Table 4). Outbreaks associated with GII.4 variants as compared to non-GII.4 variant were significantly more common in the settings of hospital acute care (p<0.01, Chi square test with Bonferroni correction) while outbreaks associated with non GII.4 variants were significantly more common in the settings of school and community social events (p<0.005, Fisher Exact test with Bonferroni correction). Most of the outbreaks in hospital acute care settings were related to GII.2006b (n = 51, 55.4%) and GII.2004 (n = 21, 22.8%).

**Table 4:** The type of settings of outbreaks caused by GII.4 variants and non-GII.4 variants.

Outbreak settings	Norovirus outbreaks from GII.4 variants	Norovirus outbreaks from Non-GII.4 variants
Community-based group residence	427 (69.2%)	56 (62.2%)
Hospital acute care	92 (14.9%)	2 (2.2%)
Hospital long term care	44 (7.1%)	2 (2.2%)
School	8 (1.3%)	8 (8.9%)
Community social event	22 (3.6%)	7 (7.8%)
Community shelter/service	6 (0.1%)	3 (3.3%)
Cruise/hotel	6 (0.1%)	3 (3.3%)
Daycare	4 (0.1%)	5 (5.5%)
Private household	4 (0.1%)	3 (3.3%)
Region-based community	2 (0.03%)	1 (1.1%)
Hospital (setting not defined)	1 (0.002%)	0 (0)
Other	1 (0.002%)	0 (0)
Total	617	90

## Discussion

By defining the annual observation period in our study in a unique way rather than by calendar year as in other studies [17], [19], [32], and access to a unique population-based dataset spanning eight years of gastrointestinal outbreaks investigated using standardized protocols allowed us to clearly observe a distinct pattern of alternating epidemic and quiescent years of NoV outbreak activity in Alberta and the northern Territories. This pattern was temporally associated with the emergence of distinctly different GII.4 variant strains. A persistent and temporal genetic drift in NoV capsid sequences was also reported worldwide [17], [27], [32]–[35]. The reason for the frequent antigenic drift of GII.4 strains resulting in critical amino acids changes (antigenic drift) in the P2 domain of the viral capsid protein binding site [35]–[38] is not understood.

Lindesmith et al. studied the molecular mechanism of GII.4 NoV evolution resulting in the persistence and emergence of new strains [21]. The emergent variants appear to have a transmissibility advantage and increased virulence as documented by the high prevalence of NoV outbreaks caused by variant 2006b/a and the steady increase in the number of NoV outbreaks over the 8 years of this study. It has been hypothesized that GII.4 NoV persist by altering their ABH histo-blood group antigens (HBGAs) carbohydrate-binding targets over time, allowing for escape from host susceptibility alleles that are highly penetrant.

An alternate explanation is that evolving strains drift under immune selective pressure until mutations have accumulated to the point where a novel genetic variant phenotype becomes established and evade from pre-established host immunity [35]. A similar phylodynamic pattern of epochal evolution has been described for influenza A virus (H3N2) [39]. Short-term protective antibodies against NoV previously described usually waned after 6 months in the absence of re-exposure [40]–[42]. Some new variants persisted for a long period of time at a low level which could be explained by ongoing mutations that allowed partial but incomplete evasion of the host immune response but did not result in increased virulence or circulation in a totally immunologically naïve population that would result in activity levels of epidemic proportion. Further studies on the immune response to NoV within populations and the duration of protective immunity against prevalent genetic variants are urgently needed.

Early detection of novel GII.4 variants could be used to forecast the NoV outbreak burden and allows enhanced surveillance and early implementation of preventive measures in populations and settings where outbreaks would most likely occur. We observed a time lag of 4 to 6 months between the initial circulation of new variants and the outbreak peak in the subsequent epidemic year. This would be the available time frame for the development of future vaccine if such capacity becomes available for NoV.

We demonstrated clearly that a single novel variant of NoV GII.4 caused the majority of outbreaks in four epidemic years in this study, similar to other reports with variable periods of observation from different continents [17], [27], [32]–[35]. However, the high prevalence of NoV outbreaks in the epidemic year 06–07 was caused by two of GII.4 variants that had emerged simultaneously (2006a and 2006b) and the total number of NoV outbreaks observed was higher than those identified in previous epidemic years. The global increase in NoV outbreaks in 2006 was linked to these two new variants [19], [20]. In contrast with the data from New Zealand and Australia [20] but consistent with observations from studies reported in the US [4] Netherlands [19] and Eastern Spain [17], the 2006b variant caused significantly more outbreaks than the 2006a variant during this period in Alberta. Siebenga et al. studied the nucleic acid and protein structure of the P2 domain of NoV capsid protein in epidemic NoV variants that emerged between 1996 and 2006 [35]. They found that the 2006a variant resulted in 8 amino acid substitutions whereas the 2006b variant had 25 amino acid substitutions in the capsid protein when compared to its predecessor, the 2004 variant. The genetic lineage of the 2006b variant was closer to the 2002 variant than to the 2004 variant in the polymerase gene region. Our results showed that genetic lineage of the 2006b was derived from the 1996 variant, whereas the 2006a variant was closer to the 2004 variant based on alignment in the E region of the capsid gene. The 2006b variant appears to have emerged from an earlier strain that had accumulated more mutations after a long period of stasis. We hypothesize that an epochal change occurred in the 2006b variant resulting in a significant number of amino acid substitutions leading to a distinct phenotype with better host evasion capability and higher transmissibility. 2006 was the first time during our observation period that two new variants emerged during the same year. Ongoing surveillance of the circulatory pattern of these strains would be critical to our understanding of the dynamics of co-circulation of NoV variants.

As observed in all NoV outbreaks worldwide [42], most of the NoV outbreaks occurred in closed settings in Alberta such as community-based group residence and hospital long term care facilities. The non-GII.4 variant were found to be significantly associated with outbreaks in in schools and community social events as compared to GII.4 variants. The number of outbreaks per observation in these two settings were small but consistent during the study period (data not shown) making the observation unlikely to be a result of temporal bias. In a previous study, we reported a broader range of NoV genotypes in sporadic gastroenteritis in young children as compared to outbreaks [31]. This study further suggested that non-GII.4 genotypes were more common in outbreaks in the younger populations. The finding might reflect differences of age-related susceptibility to different NoV stains. The population in hospital acute care was at higher risk for NoV outbreaks cause by novel variants and could be an important target for molecular surveillance for new strains.

Our study supported the importance of ongoing surveillance of circulating NoV strains at a phylogenetic level with the observation that the emergence of new GII.4 variants is associated with increase in the NoV outbreak burden. Such surveillance data has the potential to enhance public health preparedness and allow planning for more appropriate health care resource allocation. Further studies are needed to better understand the relationship between the host response and emergence of new variant strains and the dynamics of strain circulation within populations both locally and globally.
